# Spatiotemporal pattern of coastal water pollution and its driving factors: implications for improving water environment along Hainan Island, China

**DOI:** 10.3389/fmicb.2024.1383882

**Published:** 2024-04-03

**Authors:** Yunxia Du, Zhibin Ren, Yingping Zhong, Jinping Zhang, Qin Song

**Affiliations:** ^1^School of Geography and Environmental Sciences, Hainan Normal University, Haikou, China; ^2^Key Laboratory of Tropical Island Land Surface Processes and Environmental Changes of Hainan Province, Haikou, China; ^3^Northeast Institute of Geography and Agroecology, Chinese Academy of Sciences, Changchun, China

**Keywords:** coastal water, water quality, WQI model, pollution source, deterioration risk

## Abstract

In the context of human activities and climate change, the gradual degradation of coastal water quality seriously threatens the balance of coastal and marine ecosystems. However, the spatiotemporal patterns of coastal water quality and its driving factors were still not well understood. Based on 31 water quality parameters from 2015 to 2020, a new approach of optimizing water quality index (WQI) model was proposed to quantitatively assess the spatial and temporal water quality along tropical Hainan Island, China. In addition, pollution sources were further identified by factor analysis and the effects of pollution source on water quality was finally quantitatively in our study. The results showed that the average water quality was moderate. Water quality at 86.36% of the monitoring stations was good while 13.53% of the monitoring stations has bad or very bad water quality. Besides, the coastal water quality had spatial and seasonal variation, along Hainan Island, China. The water quality at “bad” level was mainly appeared in the coastal waters along large cities (Haikou and Sanya) and some aquaculture regions. Seasonally, the average water quality in March, October and November was worse than in other months. Factor analysis revealed that water quality in this region was mostly affected by urbanization, planting and breeding factor, industrial factor, and they played the different role in different coastal zones. Waters at 10.23% of monitoring stations were at the greatest risk of deterioration due to severe pressure from environmental factors. Our study has significant important references for improving water quality and managing coastal water environment.

## 1 Introduction

Coastal waters as a complex, sensitive, and highly dynamic ecosystem are strongly influenced by land-sea interactions, natural change and usage characteristics of the adjacent land (Wells et al., 2015; Chen et al., 2019; Román et al., 2023). Coastal waters have ecological importance because they can support 25% of primary production and 80% of carbon production for global scale (United Nations Environment Programme, 1992). It is confirmed that more than 60% of population and two-thirds of large and medium-sized cities in the world are concentrated along coastal areas (Ma et al., 2010; Lv et al., 2016) and most of the population live within 100 km of the coast (Gunnerson and French, 1996). Therefore, the coastal ecosystems offer important support in sustaining major socioeconomic activities, such as tourism, agriculture, and fisheries (Gai et al., 2022). However, coastal waters are therefore influenced by anthropogenic activities directly and significantly (Howard et al., 2016; Malone and Newton, 2020). In recent decades, eutrophication occurs frequently due to various human activities in the coastal area, such as the discharge of urban and industrial wastewaters, inflow of agricultural and atmospheric pollution (Bužančić et al., 2016; Cheng et al., 2020). Since the late 1980s, the rapid expansion of aquaculture in China’s coastal areas has promoted the economic development of coastal areas (Ren et al., 2019). At the same time, coastal waters became subject to intense pressure from aquaculture expansion, which inevitably leads to water quality degradation (Wang et al., 2023; Zhang et al., 2023), thus threatening the survival of aquatic life and damaging marine ecosystems. An overview about the historical variations in water quality and pollutant origins in the coastal seas of China during the 1990s and 2000s by Wang et al. (2011), indicated that water quality had not improved significantly over these two decades. Xin et al. (2023) reviewed long-term variations in pollutant sources and water quality in China’s coastal waters over the last three decades and the results showed that a turning point in the water quality appears in the mid-2010s, that is, since 2015, there has been a substantial improvement in water quality in China’s coastal waters due to the enforcement of the strictest ever Environmental Protection Law. Zhang et al. (2022) also confirmed that coastal water quality in China is improving. However, the dynamics of sensitive coastal water quality should be vigilant at all times under the pressure of enormous socio-economic activities. Sustainable management of water resource has become a challenge of critical importance.

In many countries, a range of policies and guidelines have been adopted to manage water quality and protect ecosystem in order to reverse water quality degradation. The Water Framework Directive (WFD) adopted by Member states of the European Union in 2000 was an effective instrument for the water quality management (Zotou et al., 2018). The WFD and other similar frameworks for water quality management rely mostly on collected datasets to assess water quality. In recent years, many tools and techniques have been developed to assess water quality, the water quality index (WQI) models are one of the most widely used tools (Sun et al., 2016; Sutadian et al., 2018; Uddin et al., 2020, 2022, 2023a,b; Lukhabi et al., 2023). WQI is a tool that describes the overall water quality by converting available water quality parameter data into a single unitless number by mathematical algorithms (Horton, 1965). The method is relatively easy to apply and its results are easy to interpret by both professionals and nonexperts (Uddin et al., 2021; Lukhabi et al., 2023). WQI allows water quality status to be compared across time and space, thus directly communicating information of water quality to the public and decision makers (Poonam et al., 2013; Lukhabi et al., 2023). However, several studies have indicated that there was significant uncertainty in determining the actual water quality due to the fuzziness of WQI model in structure (Kannel et al., 2007; Uddin et al., 2021; Lukhabi et al., 2023). Most of the WQI models are region-specific because their components have been developed based on expert views and local guidelines (Uddin et al., 2021, 2023c). Therefore, how to optimize water quality index (WQI) model to quantitatively assess the water quality on a large temporal and spatial scales is still not well understood.

Although several WQI models have been used to assess water quality in some lakes, rivers, and coastal waters in China (Liu et al., 2011; Hou et al., 2016; Ma et al., 2020). There is still a lack of understanding of the coastal water quality in tropical Hainan Island, China. Coastal waters are the important coastal ecosystem in Hainan Island and are also of particular interest due to their recreational, economic, and ecological values. Human pressures such as aquaculture expansion, tourism development and urban and industrial pollution could pose great risks to the deterioration of water quality and threaten the ecological security of coastal waters. The aim of this research is (1) to propose a new approach to improve the WQI model; (2) to determine spatiotemporal patterns in water quality along tropical Hainan Island by considering the specific parameters; (3) to quantify the pressures from potential environment factors to reveal the deterioration risk of water quality. This research is essential for coastal waters management and pollution control in tropical coastal areas.

## 2 Materials and methods

### 2.1 Study area

**Figure 1:**
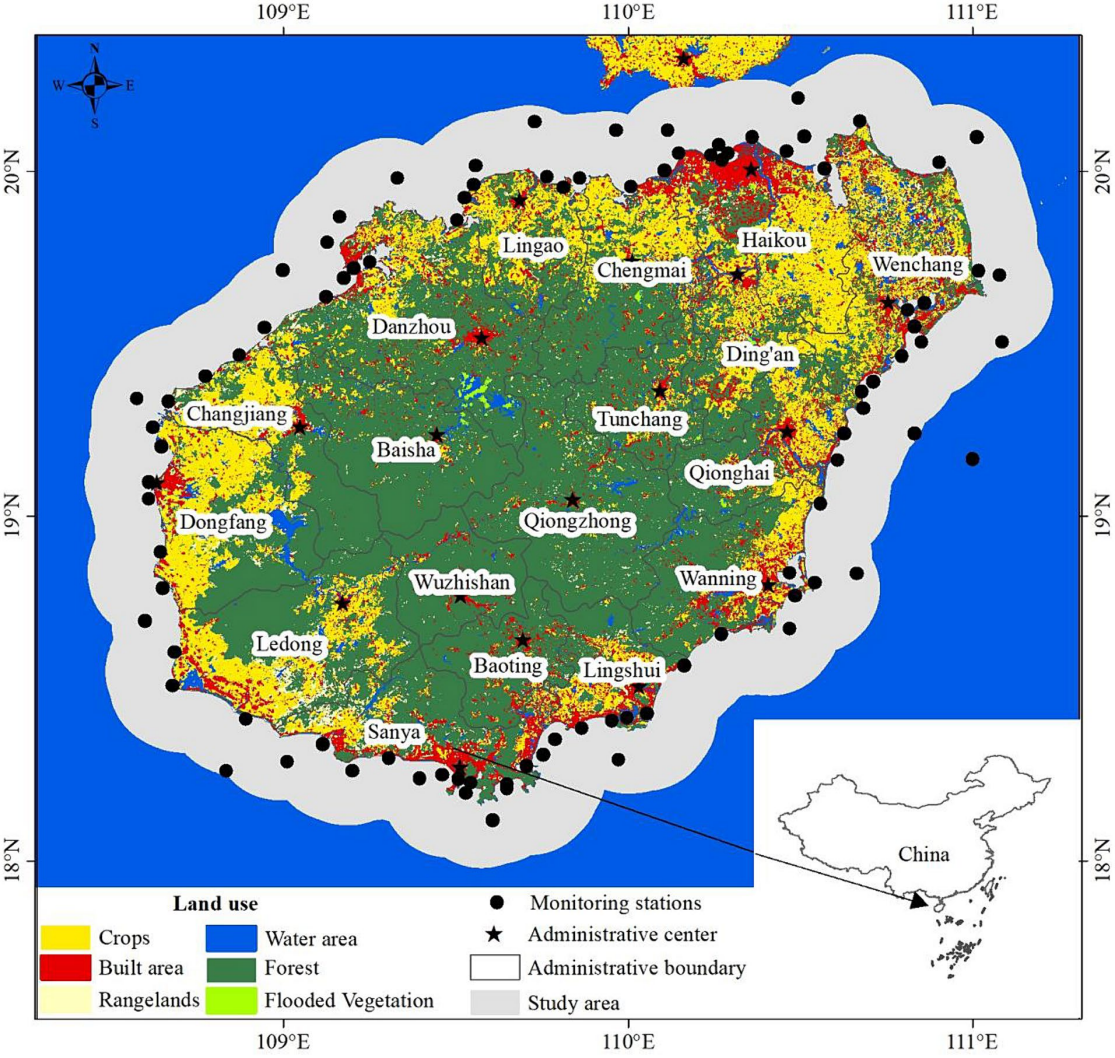
The study area and distribution of the monitoring stations for water quality.

Hainan Island (108° 37′E - 111° 03′E, 18° 10’N - 20° 10’N) is the main island of Hainan Province, China. Hainan Island is surrounded by sea and located in the south of China, facing Guangdong Province on the north, adjoining to the Beibu Gulf and facing Vietnam on the west, bordering the South China Sea on the east, adjoining to the South China Sea on the southeast and south and bordering the Philippines, Brunei and Malaysia. Hainan Island is an elliptic island with a long axis of about 290 kilometers from northeast to southwest and a short axis of about 180 kilometers from northwest to southeast. It covers an area of about 33,900 km^2^ and is the second largest island of China. The precipitation and temperature in Hainan Island show significant spatiotemporal differences, and the vegetation cover in the coastal land area of Hainan Island is unbalanced between regions. Hainan Island is rich in water resources, with 154 rivers flowing into the sea, the main rivers are Nandu River, Changhua River and Wanquan River. As a result, many estuaries and bays were formed. The coastal land of Hainan Island is an important place for human life, amusement, and production activities. Many aquaculture ponds and agricultural land are also distributed on the coastal land of Hainan Island. The water quality monitoring stations are widely distributed offshore within 20 km distance from the coastline. Therefore, the 20 km of sea along the coastline of Hainan Island was selected as the study area (Figure 1) to analyze the nearshore water environment.

### 2.2 *In situ* data and processing

*In situ* data were collected from water quality monitoring stations in offshore waters from 2015 to 2020. The frequency of water quality monitoring was generally 3 times a year, and the sampling time is arranged in March to May, July to August, and September to November. The interval between two monitoring sessions was more than 2 months. A total of 1,072 *in situ* measurements were collected from a total of 125 different stations distributed in the study area (Figure 1). A total of 39 parameters were collected, including physical, chemical, biological, and toxic substances (Table 1). Chl-a analysis and quality control were carried out in accordance with the relevant requirements of “Technical Specification for Environmental Monitoring of Offshore Waters,” Part VI: Biological Monitoring of Offshore Waters (HJ 442. 6). Other parameters were determined in accordance with “Technical Specification for Environmental Monitoring of Offshore Waters,” Part III: Water Quality Monitoring of Offshore Waters (HJ 442. 3). The *in situ* data were normalized using min-max normalization, which were then used for the correlation and driving analysis.

**Table 1:** Water quality parameters and statistics of multi-annual mean of water quality parameters collected from 2015 to 2020.

Parameter/Pronoun		Min	Mean	Max	Parameter/Pronoun		Min	Mean	Max
Temperature	T	26.	28.112	30.273	Cadmium	Cd	0.00	0.0001	0.0002
Salinity	S	10.991	31.449	34.962	Arsenic	As	0.0003	0.0012	0.003
Total suspended solids	TSS	2.827	7.268	15.136	Zinc	Zn	0.0018	0.0074	0.0136
Dissolved oxygen	DO	6.175	6.701	7.628	Chromium (VI)	Cr	0.00	0.00	0.00
pH	pH	7.893	8.115	8.2	Total chromium	T-Cr	0.00	0.0006	0.0022
Chlorophyll a	Chl-a	0.1	1.997	24.4	Selenium	Se	0.00	0.0001	0.0008
Total nitrogen	TN	0.00	0.233	1.42	Nickel	Ni	0.00	0.0007	0.0016
Total phosphorus	TP	0.00	0.012	0.037	Coliform	CB	0.00	326.17	4999.333
Transparency	SDD	1.25	2.947	8.95	Fecal coliform	FCB	0.00	281.695	4879.167
Active phosphate	AP	0.002	0.007	0.053	Biochemical Oxygen Demand in 5 days	BOD_5_	0.00	0.052	2.04
Chemical oxygen demand	COD	0.324	0.826	2.893	Cyanide	(CN)_2_	0.00	0.00	0.00
Nitrite nitrogen	NO_2_-N	0.002	0.01	0.026	Sulphide	MS	0.00	0.002	0.01
Nitrate nitrogen	NO₃-N	0.007	0.045	0.139	Volatile phenol	VP	0.00	0.00	0.00
Ammonia nitrogen	NH_3_-N	0.012	0.04	0.172	Hexachlorocyclohexane (total)	C₆H₆Cl₆	0.00	0.00	0.00
Inorganic nitrogen	IN	0.021	0.097	0.268	Clofenotane (total)	C_14_H_9_Cl_5_	0.00	0.00	0.00
Petroleum	Pe	0.0034	0.007	0.022	Malathion	C_10_H_19_O_6_PS_2_	0.00	0.00	0.00
Nonionic ammonia	N-NH_3_	0.001	0.003	0.022	Parathion-methyl	C₈HNO₅PS	0.00	0.00	0.00
Hydragenum	Hg	0.00	0.000023	0.0007	benzo[a]pyrene	C_20_H_12_	0.00	0.00	0.00
Cuprum	Cu	0.0008	0.0012	0.002	Anion surfactant	LAS	0.00	0.005	0.029
Plumbum	Pb	0.0002	0.0004	0.0011					

**Figure 2:**
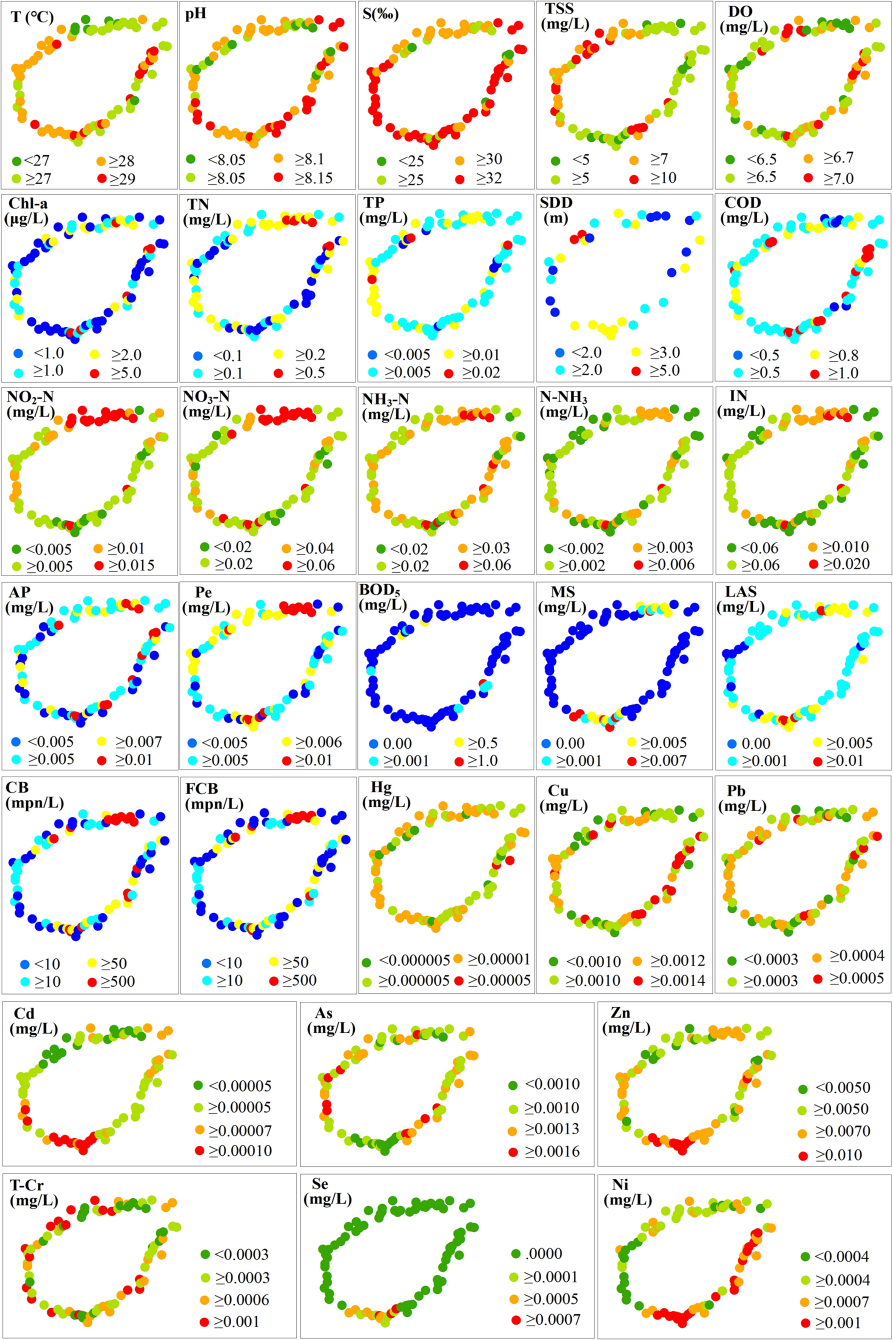
Spatial distribution of multi-annual mean of water quality parameter from 2015 to 2020.

The unit of parameters are shown in Figure 2. Pronouns for these parameters are used in the full text and in the figures.

### 2.3 Establishing an optimized WQI model for assessing water quality

The WQI model is a simple method for evaluating water quality and determining pollution levels. The WQI models is summarized into two types according to its sub-index acquisition method. The components of WQI model were determined in four main steps: (1) selecting water quality parameters; (2) computing the sub-index values; (3) assigning a weight for each parameter; (4) aggregating the sub-index value and weight of the water quality parameters (Abbasi and Abbasi, 2012; Sutadian et al., 2018). The WQI model was optimized in these four aspects, establishing an optimized WQI model in this research.

WQI tool transforms several water quality parameters into a single value and epitomized the data in a simple way (Gupta and Gupta, 2021). There were significant differences between the previous WQI models in the type and number of parameters selected and the reasons for selecting them. Parameters were typically selected based on data availability, expert opinion, or the environmental significance of a water quality parameter (Uddin et al., 2021). In our WQI model, the parameter selection ignored the type of water utilization in order to assess the comprehensive status of water quality. Parameters were selected based on the available data from monitoring stations. Total of 31 water quality parameters were applied, including not only commonly used parameters but also hazardous parameters of water quality, such as microbiological contamination, toxic compounds, and trace variables (Table 1).

Purpose of the sub-index process is to convert water quality parameter concentrations into dimensionless values (Abbasi and Abbasi, 2012). Simple linear transform function based on the measured sample range was used to obtain the sub-index. Value of the sub-index was calculated using the below Eqs 1, 2:


(1) $$C_{i}=\left(C_{\text{max}}-C_{\text{min}}\right)\times \frac{\left(X_{\text{max}}-X_{j}\right)}{\left(X_{\text{max}}-X_{\text{min}}\right)}$$

(2) $$C_{i}=\left(C_{\text{max}}-C_{\text{min}}\right)\times \frac{\left(X_{j}-X_{\text{min}}\right)}{\left(X_{\text{max}}-X_{\text{min}}\right)}$$

where *C_i_* is the sub-index value of water quality parameter *i*, which is computed for the sample *X_j_*. *C_max_* and *C_min_* are the maximum and minimum sub-index values that correspond to the maximum and minimum sample values (*X_max_* and *X_min_*) or (*X_c_* (threshold) and *X_min_*) for parameter *i*. The threshold *X_c_* is used for these parameters (such as T and DO) which have different effects on water quality depending on the concentration. The scale of the sub-index ranges between 0 and 100 (That is *C_max_* = 100 and *C_min_* = 0). Eq. 1 was used when the content of the parameter has a negative effect on the water quality otherwise Eq. 2.

In general, the parameter weight value is determined according to the relative importance of the water quality parameter or the appropriate guidelines of water quality (Sarkar and Abbasi, 2006). In our study, the unequal weighting technique was applied and the sum of weight values was equal to 1. The robustness of WQI model can be improved by using the unequal parameter weighting technology and assigning the most appropriate weighting values (Uddin et al., 2021). In order to avoid the adverse impact of inappropriate weightings on the model evaluation, an objective mathematical method based on sub-index (Eq. 3) is used for calculating weightings.


(3) $$W_{i}=\;\left(\frac{1}{N}\overset{k}{\underset{j=1}{\sum }}C_{ij}\right)/\left(\frac{1}{N}\overset{n}{\underset{i=1}{\sum }}\overset{k}{\underset{j=1}{\sum }}C_{ij}\right)$$

(4) $$\overset{n}{\underset{i=1}{\sum }}W_{i}=1$$

where *W_i_* is the weight of the i^th^ parameter. *C_ij_* is the sub-index value of the j^th^ sample of the i^th^ parameter. *N* is the number of samples for each parameter. *k* is the number of samples. *n* is the number of parameters. The weight values of all parameters must comply with the Eq. 4.

The weighted average function was used to aggregate the final WQI value as follows Eq. 5:


(5) $$WQI=\overset{n}{\underset{i=1}{\sum }}W_{i}C_{i}$$

Where *W_i_* and *C_i_* are the same as above.

### 2.4 Assessing driving factors affecting water quality

The water quality of a lake, river or ocean depends on environmental factors, including natural and human activities. In this research, water environment quality index (WEQI), which represents the pressure of natural and human factors, was used to assess water environmental factors quantitatively. The WEQI value was obtained based on an innovation to the optimized WQI model above. After parameter selection, factor analysis based on the selected water quality parameters was need to performed, and sub-index was based on the extracted factor (rather than the selected water quality parameter values). Factor analysis based on water quality parameters can determine the representative of natural and human factors, so it can solve the problem that water environmental factors are complex and variable and difficult to quantify. The calculation of sub-index, factor weighting, aggregation of sub-index and weights were still done using the above Eqs 1, 3, 5, respectively. And the weight values of all parameters still comply with the Eq. 4. The final aggregate value of the sub-index and weights of all factors was the WEQI value. The WEQI values was further classified into several levels (no pressure, slight pressure, significant pressure, extreme pressure, severe pressure) in order to assess the pressure status of water from natural and human factors. According to the pressure state from the environmental driving factors, the tendency of water quality change can be revealed.

### 2.5 Data statistics and analysis

The maximum difference (R), standard deviation (SD) and coefficient of variation (CV) of the multi-years *in situ* measurements mean between monitoring stations were calculated. The slope of the regression line is a measure of the strength of the relationship between variables, and it can represent the average changing rate of the data set. So with time as the independent variable and water quality parameter measure as the dependent variable, the slope was calculated by regression analysis and used as the changing rate of water quality parameter. A positive slope means that the parameter concentration gradually increases over time, while a negative slope means that it gradually decreases. T test by Ronald Fisher (1925) was used to test the significance. In addition, the value of these statistical indexes was spatialized to analyze their spatial distribution characteristics.

Non-parametric Spearman rank order correlations suitable for the abnormal distribution of variables were used to determine the relationship between two parameters. The correlation analysis was based on the standardized values of *in situ* water quality parameter, and the correlation coefficients (r) and statistical significance tests between parameters were automatically calculated and conducted through the Matlab 2018a software platform. Some sample values that were not tested were ignored as null values. The values of correlation coefficients were in the interval (−1 and 1), where greater than zero indicated positive correlation and less than zero indicated negative correlation.

Factor analysis was used to objectively determine relationships among parameters variables, finding the underlying factors. The KMO (Kaiser-Meyer-Olkin) test and Bartlett’s test of sphericity were used to determine whether a variable was suitable for factor analysis. The principal components analysis was used as the extraction method, with the Kaiser criterion (1960) used to guide factor selection. A varimax raw rotation was used to obtain the factor model. Significant factor loading values 0.6 were used to identify the most important variables describing the extracted factors.

The k-means clustering algorithm was used to determine the similarity between the monitoring stations considering the extracted factor scores, and then the study area was divided into different water environment zones. The clustering performance was evaluated by contour coefficient.

## 3 Results

### 3.1 Spatiotemporal patterns of water physicochemical properties

The 8 water quality parameters (Cr, (CN)_2_, VP, C₆H₆Cl₆, C_14_H_9_C_l5_, C_10_H_19_O_6_PS_2_, C₈HNO₅PS, C_20_H_12_) were excluded in the following statistics and analysis because their values were 0 (bold in Table 1). Figure 2 showed the spatial distribution of multi-annual mean of water quality parameter from 2015 to 2020. The distribution of some parameters showed significant differences along coastal regions, such as NO_2_-N, NO_3_-N, IN, Pe, CB and FCB were higher in the northern coastal region (near HaiKou) than in other regions. The lower value of T was mainly distributed in the northern coastal region. TSS showed lower values in the northern, eastern, and southern coastal regions. The higher value of DO was mainly concentrated in the northern coastal area of LinGao and the eastern coastal area of WenChang. BOD_5_ was generally low (> 0.001 mg/L at a few stations in the east and west). MS was higher in the north (near Haikou) and the south (near Sanya) and lower in the east and west. Cd, Zn and Se showed higher values in the south, while the low values of As were concentrated in this region. Ni was higher in the east and south than in the north and west. Other water quality parameters showed different spatial variation characteristics of non-significant high-low aggregation (Figure 2).

Statistics based on the multi-annual mean of water quality parameters were listed in Supplementary Figure S1. The maximum difference (R) and standard deviation (SD) of trace element (Cd, Hg, Se, Pb, Cu, Ni, T-Cr and As) among monitoring stations were the lowest (Supplementary Figure S1a), indicating a small dispersion degree relative to the average level. While CB and FCB had the highest R (4879.167 and 4999.333) and SD (787.542 and 738.868) (Supplementary Figure S1b). Coefficient of variation (CV) was used to compare the dispersion degree between different water quality parameters. Water quality parameters with larger spatial variability include Hg, Se, MS, LAS, TN, BOD_5_, Chl-a, FCB and CB, whose CV was greater than 100% (Supplementary Figure S1). Compared with these parameters, the spatial variability of other parameters was weak, with a CV less than 100% (Supplementary Figure S1).

**Figure 3:**
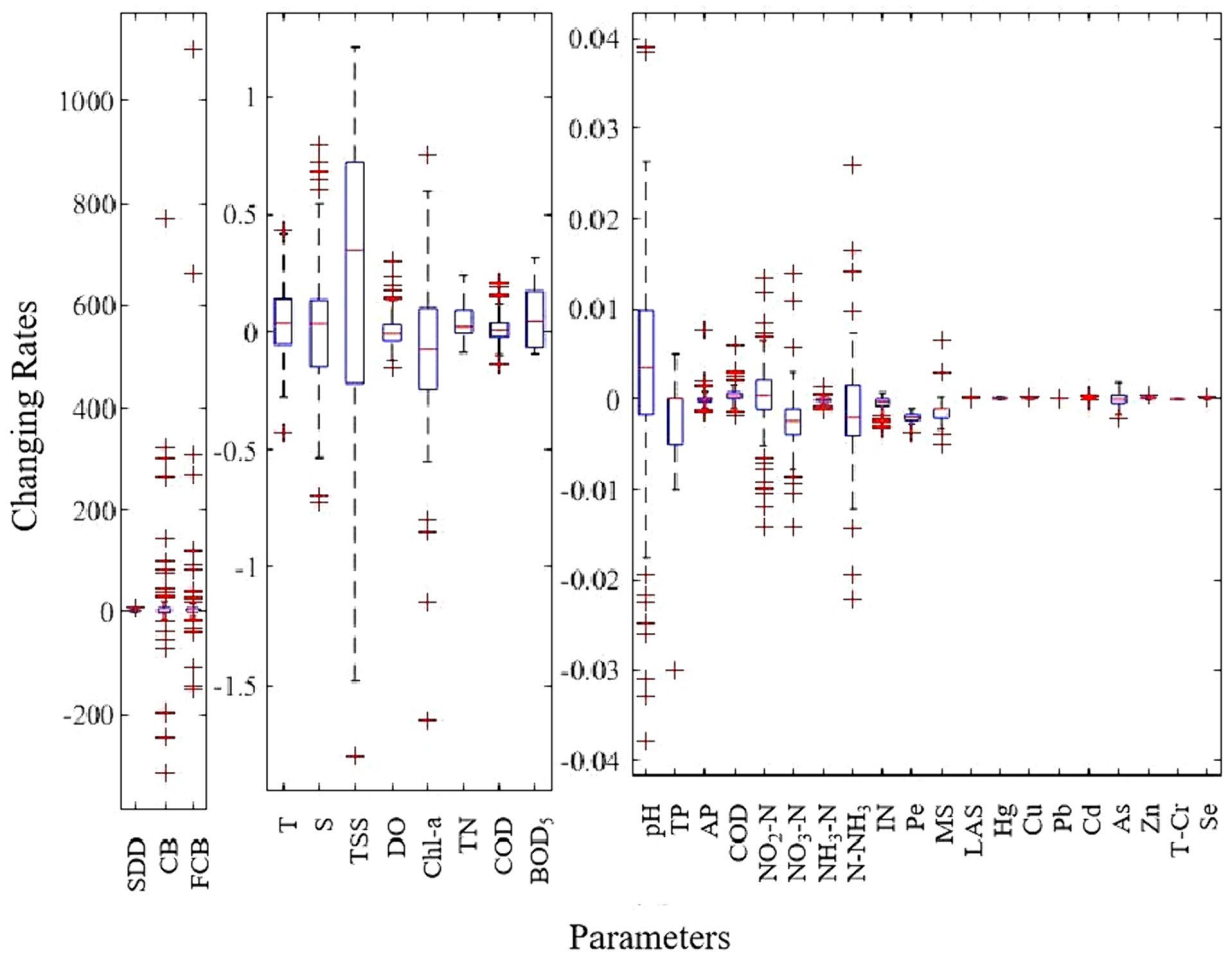
Box-plot for the changing rate of each water quality parameter at monitoring stations. Note: the units of y-axis vary depending on the parameter, “m*yr.^−1^” for SDD, “mpn/L*yr.^−1^” for CB and FCB, “°C*yr.^−1^” for T, “μg/L*yr.^−1^” for Chl-a, “yr^−1^” for pH and “mg/L*yr.^−1^” for all other parameters.

Figure 3 shows the changing rate of water quality parameters at the monitoring stations from 2015 to 2020. Overall, the changing rates of CB (−317.69—773.05) and FCB (−149.93—1104.20) had a widest range of values, followed by SDD (−1.4—9.7) (Figure 3A), indicating that the changing rates of these parameters were highly variable among monitoring stations (Supplementary Figure S2). The changing rates of T, S and TSS were significantly different among monitoring stations, and the values of changing rates for TSS at most of stations (3/4) were greater than 0 (Figure 3B), indicating that the TSS at these stations showed an increasing tendency. T generally increased in the north and southwest coastal areas (Supplementary Figure S2). S showed a decreasing tendency in the north, southwest and east (near QiongHai) (Supplementary Figure S2). TSS mainly showed an increasing tendency in the northeast and southwest (Supplementary Figure S2). The changing rates of DO ranged from −0.16 to 0.31 (more than half of the monitoring stations showed a downward tendency of DO), and DO increase mainly occurred in the north and east (Figure 3B; Supplementary Figure S2). The changing rates of Chl-a were between −1.65 to 0.75 and it decreased at 3/4 monitoring stations (Figures 3B,C; Supplementary Figure S2). The changing rates of TN, TP and BOD_5_ were generally lower (−0.09—0.24, −0.03—0.005 and − 0.095—0.32, respectively) and decreased at most monitoring stations (Figures 3B,C; Supplementary Figure S2). The changing rates of COD were between −0.13 to 0.24 (Figures 3B,C; Supplementary Figure S2). The changing rates of other parameters were generally lower and concentrated between −0.04 and 0.04 (Figure 3C).

### 3.2 Spatiotemporal assessment of water quality along Hainan Island, China

#### 3.2.1 Annual water quality assessment

Based on the annual average value of each water quality parameter collected from 2015 to 2020, the corresponding sub-index value was calculated according to the Eqs 1, 2. The sub-index value determines the size of the final water quality index value and is closely related to water quality. The weight values assigned to each parameter using Eq. 3 were listed in Supplementary Table S1.

**Figure 4:**
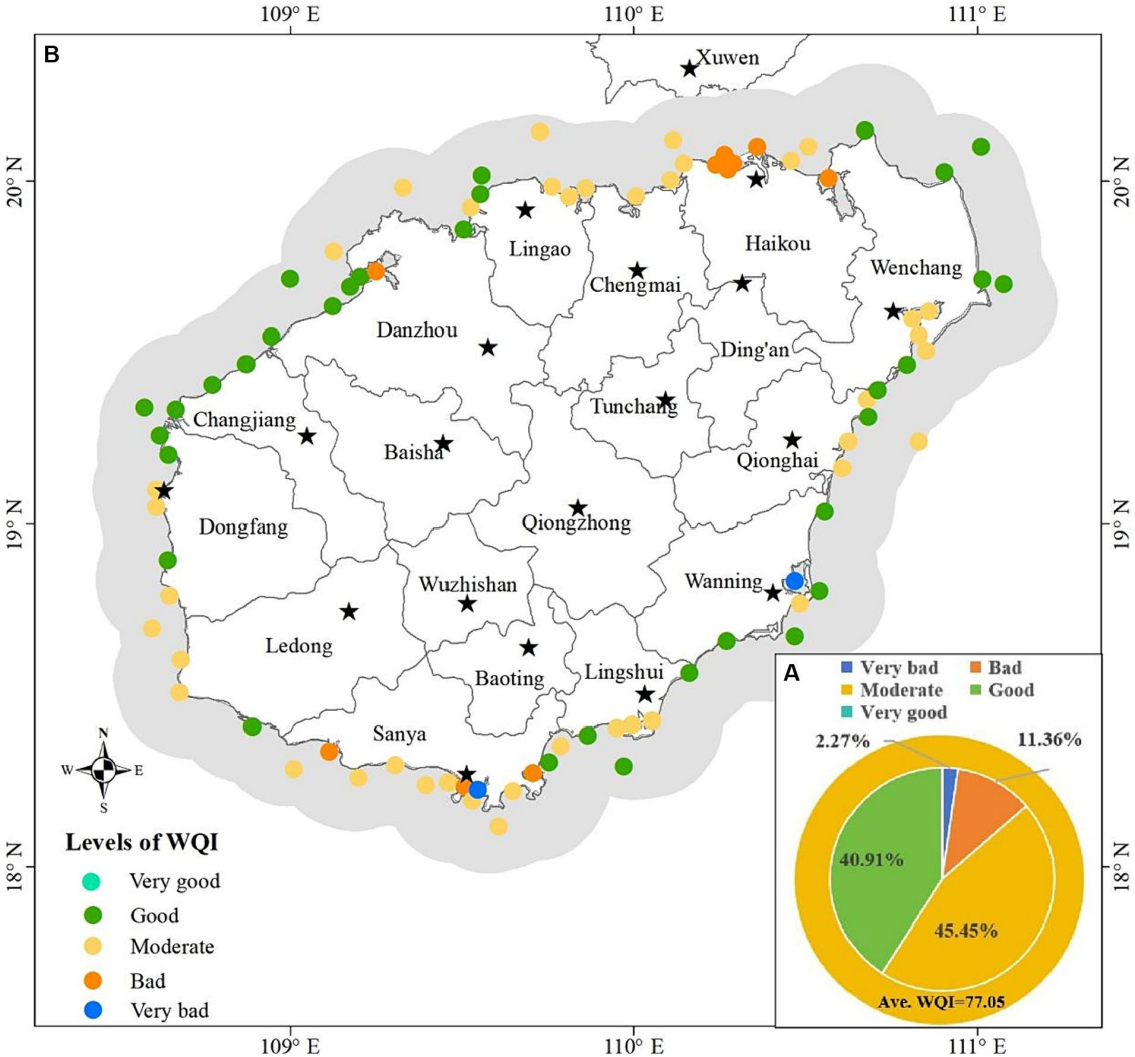
Water quality assessment based on the optimized WQI model, **(A)** average water quality and percentage at each level, **(B)** spatial variation of water quality.

Based on the aggregated WQI values by Eq. 5, five water quality levels were recommended to relatively assess the water quality in the study area, including very good (90 < WQI ≤ 100), good (80 < WQI ≤ 90), moderate (70 < WQI ≤ 80), bad (60 < WQI ≤ 70) and very bad (0 < WQI ≤ 60). The principle of this classification is to reflect the difference of water quality in the study area. Our results (Figure 4) showed that none of the monitoring stations analyzed showed the level of “Very good.” Of these stations analyzed, 40.91% showed as “Good,” 45.45% showed as “Moderate,” 11.36% showed as “Bad,” 2.27% showed as “Very bad” (Figure 4A). The average value of aggregated water quality index for the study area was 77.05, so the water quality of the study area generally showed as a moderate level (Figure 4A). Figure 4B showed the spatial distribution of water quality at each level. The average water quality status at one monitoring station in coastal water of Wanning and one in coastal water of Sanya showed “Very bad” levels. The monitoring stations with “bad” water quality level were mainly located in the coastal water of Haikou and Sanya. Monitoring stations with Moderate water quality were located in coastal waters except the Changjiang. Some coastal waters in the west and east area showed “Good” water quality.

#### 3.2.2 Seasonal water quality assessment

**Figure 5:**
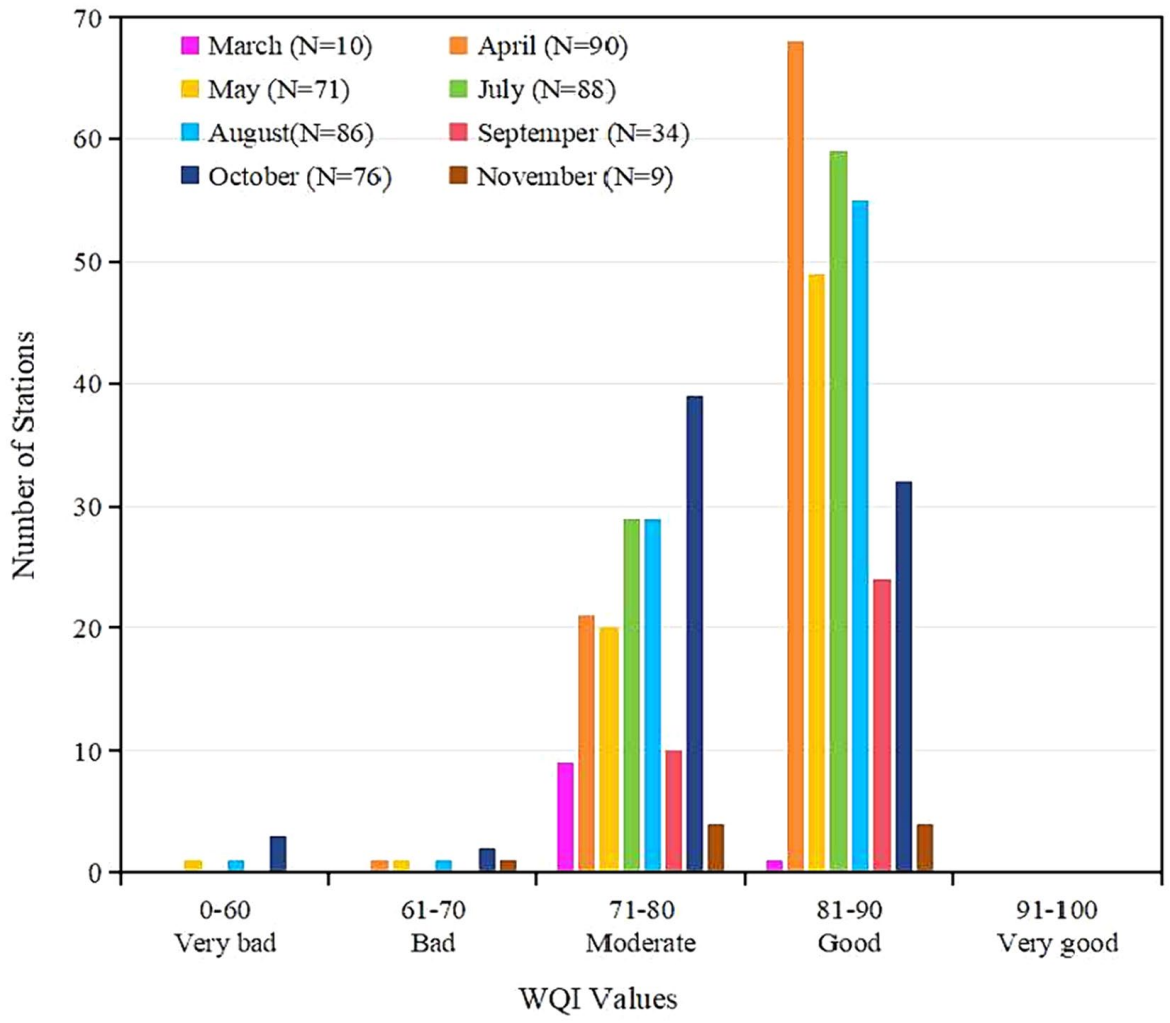
Histogram of results of seasonal water quality assessment based on the optimized WQI model. N is the number of monitoring stations analyzed.

**Figure 6:**
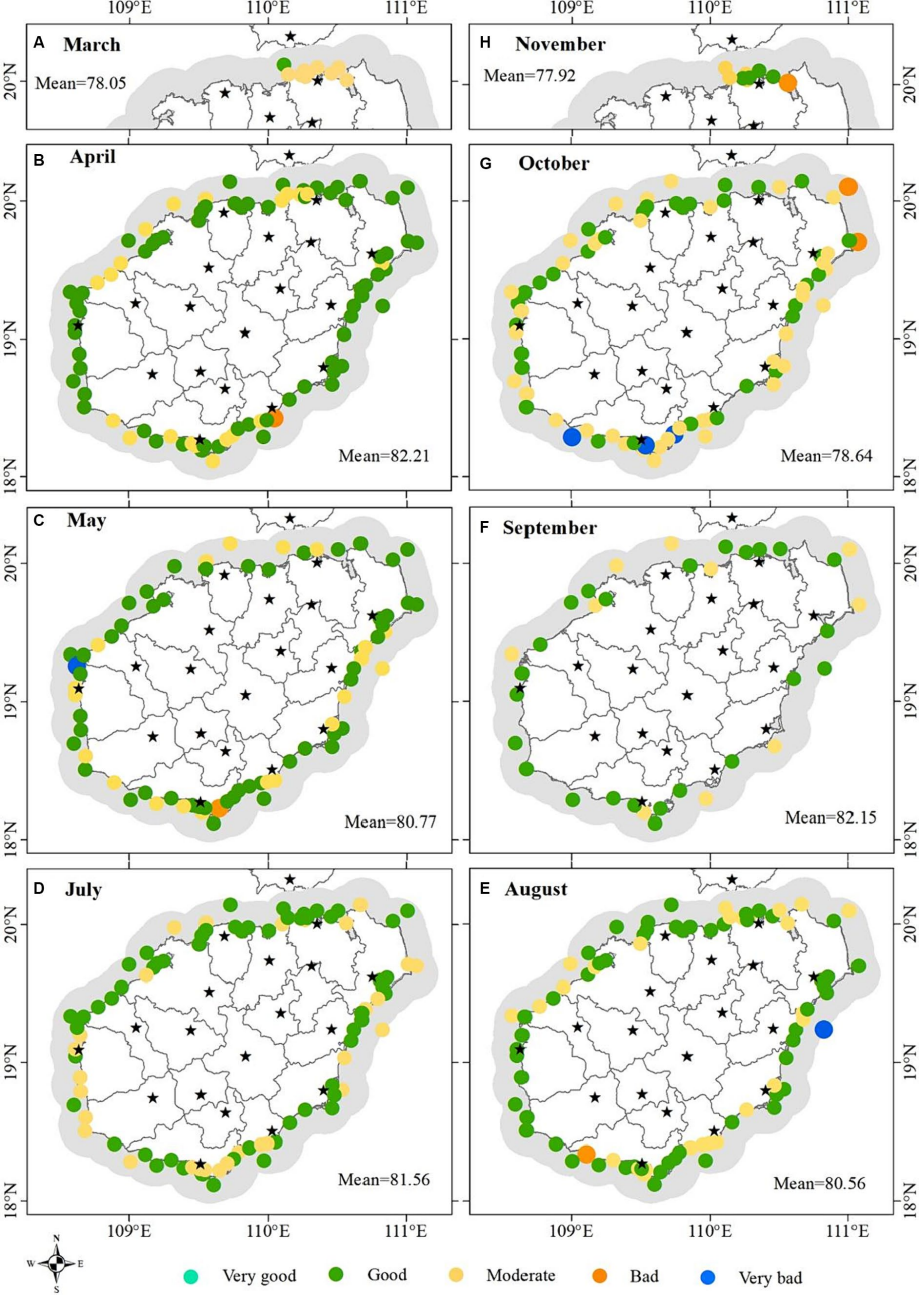
**(A–H)** Spatial distribution of water quality assessment at the analyzed monitoring stations in March, April, May, July, August, September, October, and November, respectively.

Similarly, the corresponding sub-index value was calculated according to the Eqs 1, 2 based on the average value of each parameter at the monitoring station in each month from 2015 to 2020. The aggregated WQI values by Eq. 5 was still classified as the five water quality levels recommended above. The results showed that none of the monitoring stations and none of the month (March to November)’ WQI value was at the level of “Very good”; in any given month, the WQI value at the monitoring stations analyzed was mostly at the levels of “moderate” and “good” (the proportion of monitoring stations at “Moderate” level was greater than that at “Good” level in March and October, it was the opposite in April, May, July, August and September, it was equal in November); the WQI values of a very small number of monitoring stations were at the levels of “Very bad” and “Bad” in April, May, August, October and November (Figure 5). Figure 6 showed the spatial distribution of water quality assessment at each monitoring station for each month. Overall, the averaged water quality of all monitoring stations fluctuated at “Good” level from April to September, and decreased significantly to “Moderate” level in October (Figures 6B–G). From the monitoring stations analyzed, water quality at “Very bad” level occurred in May (Figure 6C), August (Figure 6E) and October (Figure 6G). Water quality at “Bad” level occurred in April (Figure 6B), August (Figure 6E), October (Figure 6G) and November (Figure 6H).

### 3.3 Assessment of influencing factors for water quality

#### 3.3.1 Correlations between water quality parameters

**Figure 7:**
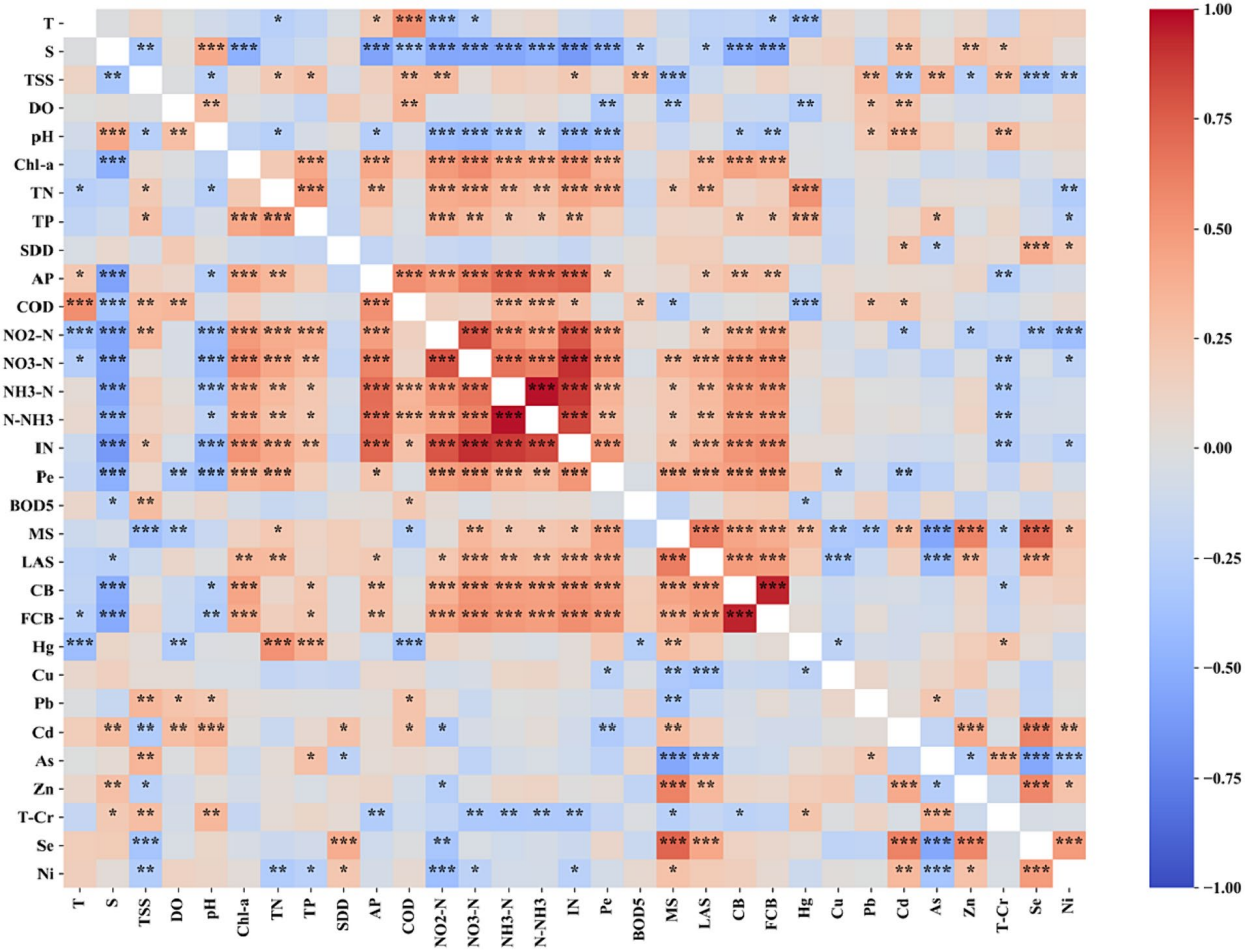
Correlation coefficient (r) and significance level (p) between water quality parameters. Note: The white blocks in the matrix represent missing r values due to no synchronized measurements; “***” is the significance level *p* < 0.001, “**” is the significance level 0.001 ≤ *p* < 0.01 and “*” is the significance level 0.01 ≤ *p* < 0.05.

In addition to the parameters (Cr, (CN)2, VP, C₆H₆Cl₆, C_14_H_9_C_l5_, C_10_H_19_O_6_PS_2_, C₈HNO₅PS, C_20_H_12_) that were not found in the 344 samples, the spearman correlation analysis significance test was performed between the simultaneous measurements of other parameters (Figure 7). A total of 259 pairs of parameters were positively correlated, while 206 pairs were negatively correlated. The significance test showed that the significance level (p) of 216 pairs of parameters was less than 0.05, that of 163 pairs was less than 0.01 and that of 117 pairs was less than 0.001. The correlation between the trace element parameters and non-trace element parameters was not universally significant. This indicates that there is no significant interaction between the two types of elements. There were significant correlations between some trace elements parameters (such as between Se, Cd, Ni, Zn). Chemical and processing enterprises are the main external sources of trace element pollution in the Hainan Island region. These trace elements may be abundant in the same material used in industry, resulting in a positive correlation between these elements. There were generally significant positive correlations between Chl-a and nutrient (NO_2_-N, NO_3_-N, NH_3_-N, N-NH_3_, IN, AP, TP, TN). This is because nutrients are the main cause of water eutrophication and cyanobacteria reproduction. Phytoplankton containing chl-a can grow rapidly when nutrients are sufficient. These nutrients also had significant positive correlation with each other. In general, there are internal and external two sources of nutrients in water. These nutrients can be converted into each other under the action of microorganisms. For example, sufficient NH3-N promotes the enrichment of NO_3_-N through digestion, and then increases the content of NO_2_-N through oxidation. On the other hand, sufficient NO_2_-N is reduced to NH_3_-N by denitrification under certain conditions, and the content of NH_3_-N is increased. It has been shown that organic matter in water has a strong stimulating effect on the formation of sulfide and organic matter in water is closely related to nutrients. Nutrients in water can promote the growth and reproduction of CB and FCB. Therefore, there was significant positive correlation between nutrients and Pe, MS, LAS, CB and FCB. In addition, these nutrients had significant negative correlation with S and pH. This may be due to the fact that a certain S and pH in the water can promote the absorption of nutrients by phytoplankton. The pH and S can kill CB and FCB, so there was a significant negative correlation between them.

#### 3.3.2 Analysis of the influencing factors for water quality

**Figure 8:**
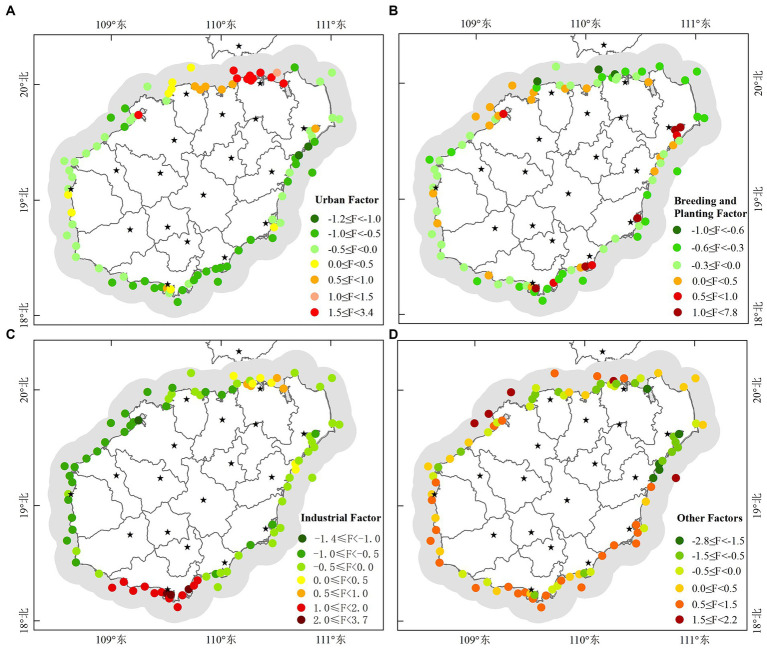
Spatial distribution of the factors extracted by factor analysis: **(A)** Urban factor (factor 1), **(B)** breeding and planting factor (factor 2), **(C)** industrial factor (factor 3) and **(D)** other factors (factor 4). The “F” in the legend represents the factor.

In order to further objectively reflect the internal relationship between the variables and find the pollution sources of water environment, factor analysis was further carried out based on the water quality parameters. The KMO value was 0.701. In addition, the *p*-value of Bartlett’s test of sphericity was 0.00, which was less than 0.05. Therefore, our data was suitable for factor analysis. Based on the initial eigenvalue (greater than 1) obtained by principal component analysis and the eigenroot gravel map (mutation point), four factors were extracted (Supplementary Table S1), which together explained 89.65% of the total variance of the water environment indexes. According to the load values of water quality parameters on the factors, factor 1, factor 2, and factor 3 were interpreted as urban factor, breeding and planting factor, and industrial factor, respectively. Factor 4 may be derived from meteorological conditions, recreational activities, etc., so it was interpreted as other factors (Supplementary Table S1). Spatial distribution of the extracted factors (Figure 8) indicated that there was variability in the influencing factors of water environment in the study area. Spatial distribution of the urban factor (Figure 8A) showed that high and intermediate values occurred in the northern region, which is close to the provincial capital (Haikou). High values of the breeding and planting factor (Figure 8B) were detected in the regions, which is close to Wenchang, Wanning, Lingshui, Sanya and Danzhou. Intermediate values were also found in other regions. According to the spatial distribution of industrial factor (Figure 8C), high values occurred in the southern region, low and intermediate values occurred in the northern region. Intermediate to high values for the other factors were widespread at most stations.

**Figure 9:**
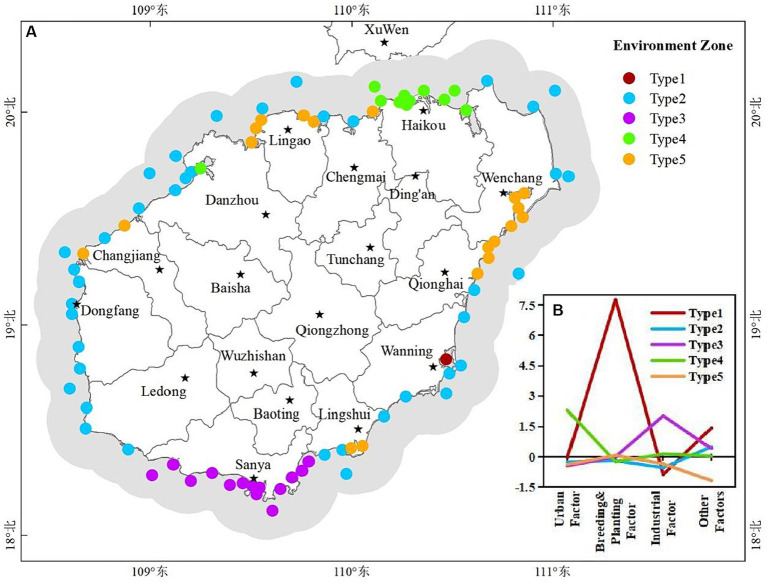
**(A)** Major environment zones for water generated by clustering based on extracted factor features. **(B)** Characteristics of pollution sources for environment zones.

According to the characteristics of extracted factor scores, monitoring stations were classified into five types of water environment zones through clustering of monitoring stations (Figure 9A), indicating that the water environment in the study area had significant spatial heterogeneity. The nearshore sea near the northern Haikou showed type 4 (Figure 9A), which was mainly affected by urban factors and is weakly affected by industrial factors (Figure 9B). The nearshore sea along Sanya in the south showed the type 3 water environment zone (Figure 9A), which was mainly affected by industrial factors (Figure 9B). In the west, the nearshore sea along Wenchang southeast and Qionghai mainly displayed as type 5 (Figure 9A), and this type of water environment zone was mainly affected by breeding and planting factor (Figure 9B). The nearshore sea along Wenchang northeast and WanNing displayed as type 2 (Figure 9A), which was mainly affected by other factors (Figure 9B). Danzhou, Dongfang and Ledong in the east mainly showed as type 2 water environment zone (Figure 9A). Lingshui in the southeast, Chengmai and Lingao in the north, and Changjiang in the west showed two types of water environment zones (type 2 and type 5). Water environment zone type 1 was located at the estuary of Wanning (Figure 9A), which was mainly influenced by breeding and planting factor, and was influenced by other factors weakly (Figure 9B).

**Figure 10:**
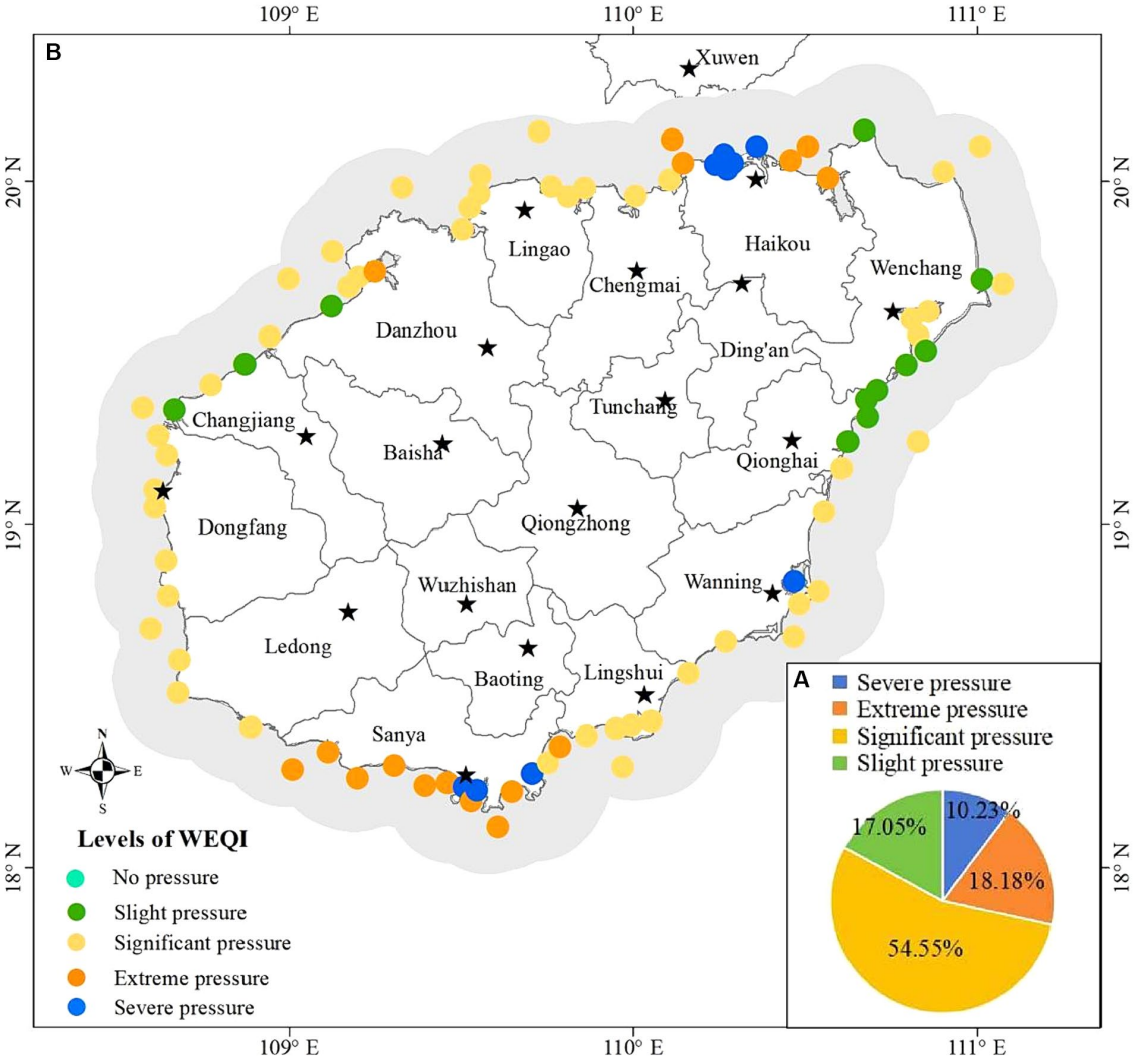
Water environmental factors assessment based on extracted factors, **(A)** Percentage of monitoring stations at each pressure level, **(B)** distribution of the pressure levels.

Environmental factors were further quantified based on the factor analysis. The WEQI values were calculated by taking the factors extracted from factor analysis as input parameters of the optimized WQI model (all factors were considered to have negative effects on water environmental quality). The weights of each factor were shown in Supplementary Table S1. The aggregated WEQI values were shown in Figure 10. The low WEQI value indicated that the water was under great pressure from environmental factors. It could be seen that 10.23% of the monitoring stations were under the level of “Severe pressure” from environmental factors, 18.18, 54.55 and 17.05% were at the pressure level of “Extreme pressure,” “Significant pressure” and “Slight pressure,” respectively (Figure 10A). Those monitoring stations at the level of “Severe pressure” were located in the coastal water of Haikou, Sanya and Wanning, and those at the level of “Extreme pressure” were in the coastal waters of Haikou, Sanya and Danzhou (Figure 10B). Some coastal waters in Wenchang, Qionghai, Danzhou and Changjiang were just under “Slight pressure” level from environmental factors (Figure 10B).

## 4 Discussion

### 4.1 The reliability of the optimized WQI model

Each of the four main processes involved in a WQI model can contribute to the model uncertainty (Gupta and Gupta, 2021; Uddin et al., 2023a,b,c). In this research, the correlation analysis between water quality parameters showed that there were significant correlations among some parameters (Figure 7). Nevertheless, the 31 available water quality parameters in our study contained different properties, including physical, chemical, biological and trace elements. This avoided the influence of insufficient selection of typical parameters on the reliability of WQI assessment results. Some parameters such as T, DO, pH should be treated differently when obtaining sub-index due to their influence on water quality is bidirectional (Le et al., 2023). In this research, the bidirectional effects of parameters on water quality were considered through using Eqs 1, 2 in the process of developing the sub-indexes, respectively. Supplementary Figure S3 showed the recommended critical values for each parameter in this research and a simplified depiction of their effects on water quality. It should be noted that the critical value should be determined according to local climatic conditions and geographical environment in practical application. A weighting strategy for parameters based on sub-index was adopted in this research (Eq. 3). Weights of the parameters depends on the values of the sub-index, which could avoid the influence of subjective factors on the reliability of the WQI model. On the premise that water quality parameters are equally important, the WQI model using this weighting strategy is objective and reliable for the evaluation of any specific data set.

Most developed WQI models were region-specific because their components were developed based on expert advice and local guidelines (Gupta et al., 2017). The components of our optimized WQI model were not developed based on expert opinions and local guidelines, and the results from this model are not comparable to those from other WQI models. The approach of optimizing WQI components in this research is applicable to other water quality assessment including lakes and rivers. The assessments obtained by the optimized WQI model can fully reflect the difference of water quality between different regions, so it can provide an effective reference for water environment governance decision makers.

### 4.2 Environment factors affecting water quality

By performing factor analysis on water quality parameters, representative environmental factors can be determined, which solves the problem that water environmental factors are complex and difficult to quantify (Flo et al., 2011; Le et al., 2023). This research was the first attempt to relate the variability of water quality to specific factors. We found that there were four major pollution sources which potentially contribute to deteriorating the quality parameters in the study area (Supplementary Table S1; Figure 8). The loading values of factors (Supplementary Table S1) showed that factor 1 was defined by NO_3_-N, IN, Pe, NO_2_-N, CB, TN, FCB, TP and pH. Domestic sewage from cities entering coastal waters can cause these parameters to increase (Flo et al., 2011; Xiao et al., 2020). The urban pollution source was also strongly supported by results in Figure 2, which showed that the value of these parameters (except the pH) was greater in the coastal waters near city (Haikou). Factor 2 had a higher loading value with AP, N-NH_3_, Chl-a, COD, S, BOD_5_, NH_3_-N and T. They could be affected by runoff carrying agricultural pollutants to coastal waters or by aquaculture effluent discharges carrying pollutants to coastal waters (Le et al., 2023). It was also supported by the characteristics of human activity in the affected areas dominated by factor 2 (Figure 9) that these parameters could be connected to the activities of planting and breeding activities. Factor 3 was defined by Se, MS, Zn, LAS, Cd, As, Ni and TSS. Industrial wastewater discharge and the operation of boats on the water often causes such pollutants in the surface water. The dominant influence area of factor 3 was the coastal waters of Sanya, where industrial facilities are distributed. Therefore, this factor was interpreted as an industrial factor. Factor 4 was defined by T-Cr, Cu, DO and Hg. Specific pollution sources for these parameters were still unclear and may need more research. SDD and Pb were not expressed by any factors. Other potential environmental factors also need to be studied further.

As confirmed in this research, coastal waters are not homogeneous but can be divided into different environment zones based on dominant factors (Figures 9A,B). Urban factors and industrial factors were demonstrated to be the main causes of pollution in coastal water of Haikou and Danzhou (type 4 in Figure 9A), in coastal water of Sanya (type 3 in Figure 9A), respectively. Planting and breeding were demonstrated to be the primary cause of pollution in coastal water of Wanning, Wenchang, Qionghai, Linggao, and Changjiang (type 1 and type 5 in Figure 9A). Therefore, in the practice of environmental management and water pollution treatment, it is necessary to take countermeasures according to the dominant influencing factors in different regions.

### 4.3 Suggestions for improving water quality

The classified WQI levels could reflect the relative differences of water quality between monitoring stations, which can be the basis for guiding water quality management decisions. This classification method is uncertain because there is no fixed standard. The level threshold needs to be determined based on all the values. Unreasonable classification is not conducive to identifying problematic waters and is not conducive to improving water quality effectively. However, this classification is sufficient to reflect relative differences of water quality between stations and has important significance to guide decisions of water quality management. Water quality near two monitoring stations located in Sanya and Wanning was at “Very bad” level (Figure 4), so it urgently needs to be improved. These two regions have higher pH, Chl-a, AP, COD, NO_3_-N, IN, NO_2_-N, CB, FCB, N-NH_3_, NH_3_-N, T and Ni according to the distribution of water quality parameters (Figure 2). Therefore, improving water quality should focus on the pollution sources of these parameters. The characteristics of environmental areas and pollution sources generated by clustering in this study (Figures 9A,B) can effectively guide the implementation of water quality management measures. According to the Figure 9, the aquaculture factor near the monitoring station in Wanning should be emphasized, that is, we can improve the water quality through reasonable management of the aquaculture industry. And it is recommended to strengthen regulation and control on industrial factors near the monitoring station in Sanya should be strengthened. Similarly, those regions where the water quality at “Bad” level (Figure 4) also need to be improved. According to the distribution of these monitoring stations and their main pollution sources in Figures 4B, 9, measures should be taken in the urban factors for the waters of those monitoring stations along Haikou and Danzhou, while the regulation on industrial factors should be strengthened for those monitoring stations along Sanya. Although the water quality of most stations analyzed in the study area was at “Good” level and “Moderate” level (Figure 4), improvements were still recommended for the parameter that were significantly high (Figure 2). From the seasonal variation of water quality, it could be seen that the water quality of some monitoring stations would be degraded in certain months (Figure 6). For example, the water quality of some monitoring stations along Sanya might deteriorate in October. Therefore, we need to take improvement measures in October for those monitoring stations.

The WEQI value (Figure 10) obtained in this research can showed the pressure status that water is suffer from environmental factors, revealing the risk of water quality deterioration. The statistics of WQI and WEQI (Table 2) showed that waters at 13.64 and 27.27% of the monitoring stations with “Good” level had slight and significant risk of deterioration, respectively; waters at 3.41, 27.27 and 14.77% of the monitoring stations with “Moderate” level showed slight, significant and extreme risk of deterioration, respectively; waters at 3.41 and 7.95% of the monitoring stations with “Bad” level showed extreme and severe risk of deterioration, respectively; waters at 2.27% of the monitoring stations with “Very bad” level showed severe risk of deterioration. Even water at the same WQI level may have different risks of deterioration due to different pressures from environmental factors. Therefore, we recommend that in practice, we should not only pay attention to those waters with poor water quality (the lower WQI levels in Figures 4, 6) but also pay attention to those waters with greater risk of deterioration (the Significant, extrem and severe pressure levels in Figure 10).

**Table 2:** Comparison between the results of water quality assessment and water environmental factors assessment.

WEQI	WQI					Total
	Very good	Good	Moderate	Bad	Very bad	
No pressure	0	0	0	0	0	0
Slight pressure	0	13.64%	3.41%	0	0	17.05%
Significant pressure	0	27.27%	27.27%	0	0	54.55%
Extreme pressure	0	0	14.77%	3.41%	0	18.18%
Severe pressure	0	0	0	7.95%	2.27%	10.23%
Total	0	40.91%	45.45%	11.26%	2.27%	100%

### 4.4 Potential hazards from water pollution

Coastal waters are important places for aquatic organisms to reproduce and multiply. Pollutants can cause serious disasters to the ecology and biodiversity of coastal waters, and also endanger human health (Zoppini et al., 2019; Chi et al., 2021). It has been reported that a decrease in DO concentration will result in a decrease in the number of aerobic bacteria (Zhao et al., 2014). DO at several stations in this research was found to be less than 6.5 mg/L (Figure 2), which is not conducive to the growth of aerobic bacteria. Liu et al. (2024) showed that chl-a, pH and phosphate-P in summer, and electric conductivity, nitrate-N and ammonium-N in winter affected the bacterial community variation. And they argued that high nitrate and ammonia nitrogen can promote nitrification and increase the abundance and diversity of bacteria. Significant nutrient pollutions were found at some stations in our research, such as high NO_2_-N (≥0.015 mg/L), NO_3_-N (≥0.06 mg/L), NH_3_-N (≥0.06 mg/L), N-NH_3_ (≥0.006 mg/L), IN (≥0.02 mg/L), TP (≥0.02 mg/L) and TN (≥0.5 mg/L) concentrations (Figure 2). These stations are therefore beneficial for nitrogen-oxidizing bacteria and these microorganisms may greatly increase to affect the balance of community structure. High nitrogen and phosphorus nutrients also can lead to eutrophication of water, resulting in cyanobacteria bloom (Bužančić et al., 2016). Several trace elements were detected in our research and several stations have relatively high levels of trace element, such as Cu (≥0.0014 mg/L), Pb (≥0.0005 mg/L), Cd (≥0.0001 mg/L), Zn (≥0.01 mg/L), As (≥0.0016 mg/L), Ni (≥0.001 mg/L), T-Cr (≥0.001 mg/L) (Figure 2). Trace elements like Fe, Cu, Zn, Ni and others are important for the proper functioning of biological systems and their deficiency or excess are detrimental for aquatic microorganisms (Aithani et al., 2023). Trace element in water is thought to be difficult to degrade, bioaccumulative and ecologically toxic (Wu et al., 2016; Saha and Paul, 2019). Aquatic trace element could be absorbed in large quantities by microorganisms and affect terrestrial organisms or humans through biological enrichment, food chain amplification and other pathways (Oropesa et al., 2017). High toxic trace elements in the water may poison some microbes and those heavy metal-resistant (e.g., Acidobacteriota) may increase (Zhang et al., 2024). *Escherichia coli* (CB) and fecal *Escherichia coli* (FCB) in our study are used as microbial indicators of water quality in water quality assessment. CB and FCB in water can seriously harm human health through the food chain (Reynolds et al., 2008; Hu, 2024). Several stations in this research had CB and FCB levels in excess of 500mpn/L, posing a potential risk of outbreak. Outbreaks of these community may result in serious consequences, such as diarrheal disease, which can even lead to death (Reynolds et al., 2008). However, there is still a lack of in-depth research on the relationship between pollution and microbial community and human health, which will be a meaningful content for future research directions.

## 5 Conclusion

Based on a datasets of 31 water quality parameters collected from 2015 to 2020, an optimized WQI model was developed to assess spatial and temporal water quality. Pollution sources of water quality were also identified by factor analysis and its deterioration risk was further assessed using the index WEQI. The following conclusions can be made:

(1) The uncertainty of water quality assessment can be weakened by the optimization of WQI model process. The approach of optimizing WQI model in our research is applicable to water quality assessment of other regions (including lakes and rivers) or other datasets.
(2) The average water quality was at moderate level. The water quality of this region showed temporal and spatial variability. Water quality at 13.53% of the monitoring stations was relatively poor (“Bad” level or“Very bad” level) and they were mainly appeared in the coastal waters of large cities (Haikou and Sanya) and in some aquaculture waters. Average water quality in March, October and November was worse than in other months.
(3) Water quality of this region was affected by at least four main pollution sources including urban factor, planting and breeding factor, industrial factor and other factors. These factors play a different dominant role in different regions, respectively. Waters at 10.23% of monitoring stations were at the greatest risk of deterioration due to severe pressure from these environmental factors.

## Data availability statement

The original contributions presented in the study are included in the article/Supplementary material, further inquiries can be directed to the corresponding author.

## Author contributions

YD: Conceptualization, Data curation, Funding acquisition, Methodology, Software, Supervision, Writing – original draft. ZR: Conceptualization, Methodology, Supervision, Validation, Writing – review & editing. YZ: Writing – review & editing, Investigation, Formal analysis. JZ: Writing – review & editing, Formal analysis, Data curation. QS: Formal analysis, Investigation, Validation, Writing – review & editing.
