# Supplementary material for: Spatiotemporal pattern of coastal water pollution and its driving factors: implications for improving water environment along Hainan Island, China
Source: Front Microbiol. 2024 Apr 3;15:1383882. doi: 10.3389/fmicb.2024.1383882 (PMC11021667; doi:10.3389/fmicb.2024.1383882)
Supplement: Supplementary file 1 [file Table_1.DOCX]

**Supplementary Material**


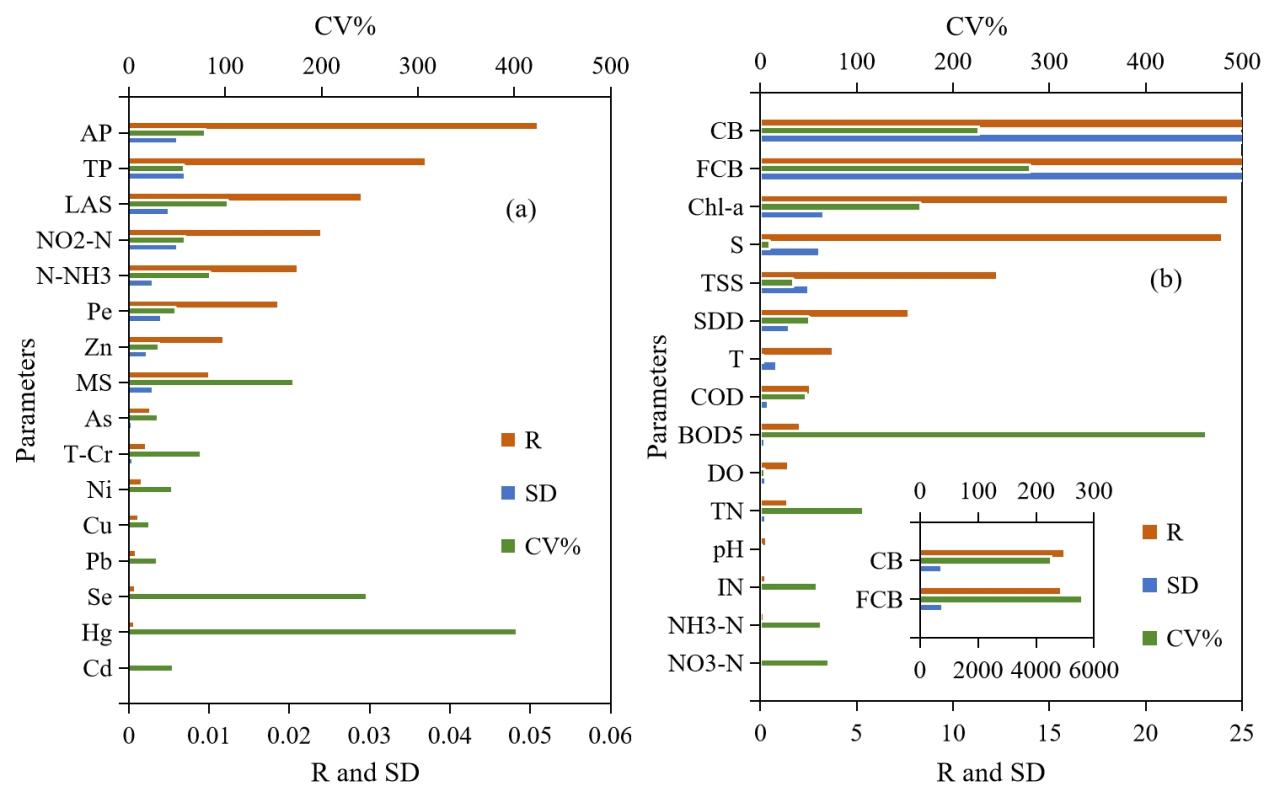


Fig. S1 Statistics of water quality parameters at monitoring stations. R is maximum difference between the maximum and the minimum values. SD is standard deviation, CV is coefficient of variation.


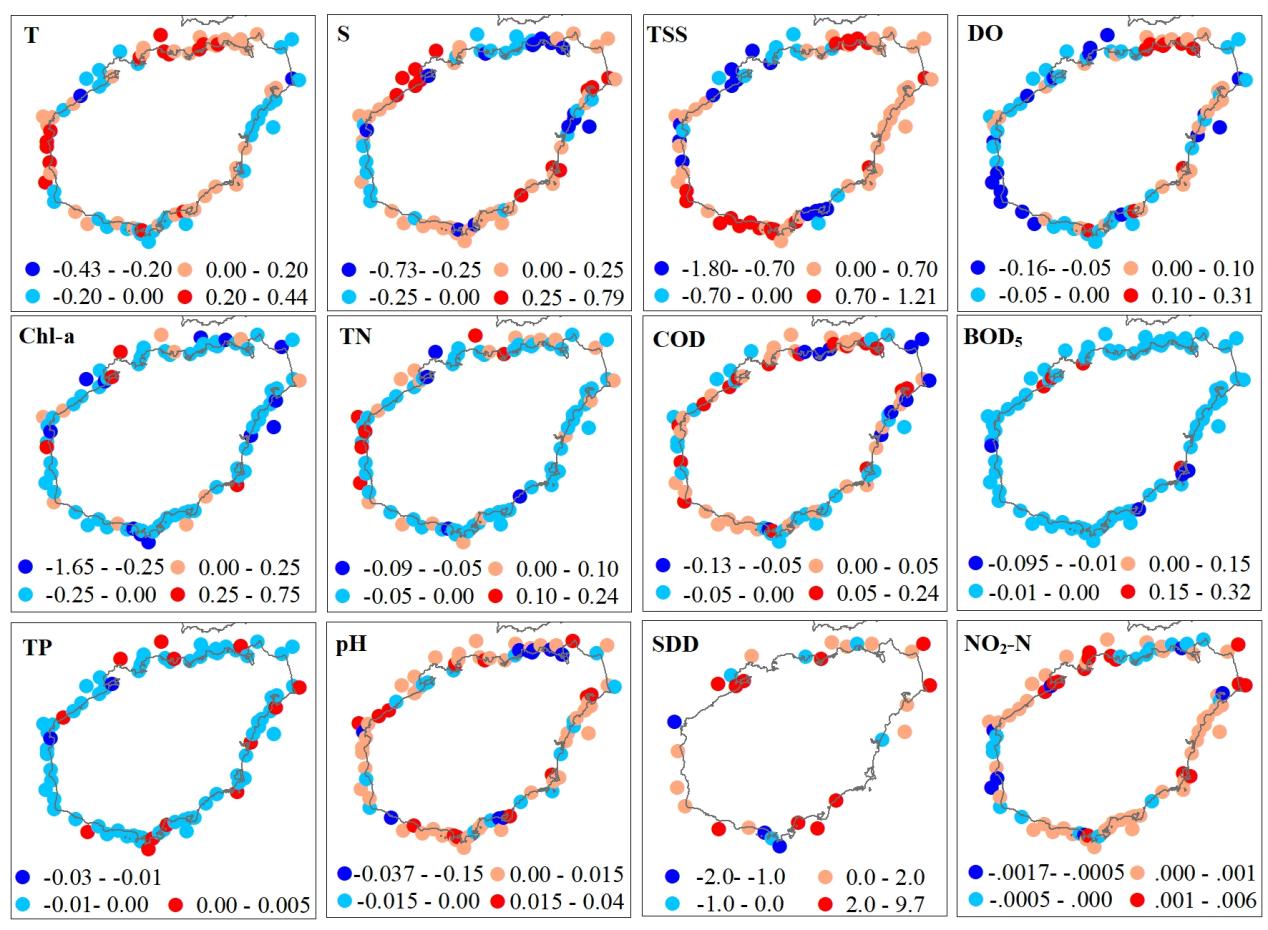


Fig. S2 Spatial distributions of the changing rates for water quality parameters at monitoring stations. Note: Only the parameters for which the changing rates varied significantly among monitoring stations of shown here.


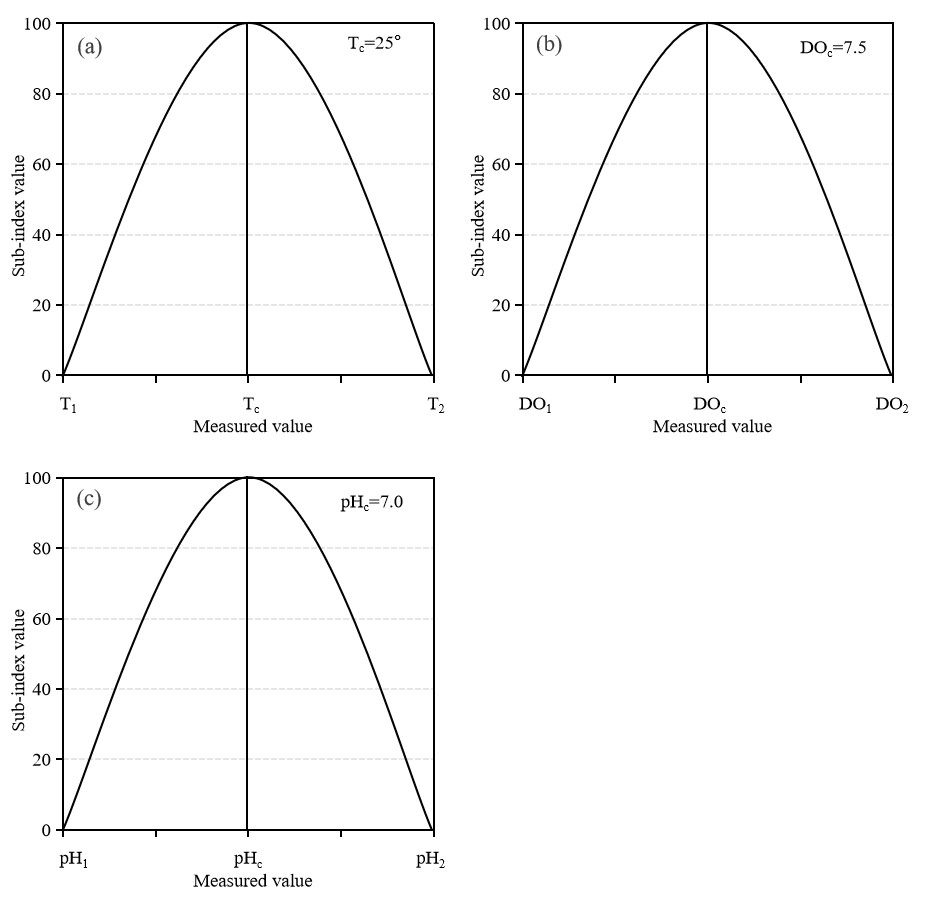


Fig. S3 Recommended critical values and simplified relationships between parameter measured values and corresponding sub-index values.

Table S1 Loading values of water quality parameters derived from factor analysis, weight values of the parameters used to calculate WQI and weight values used to calculate WEQI factors derived from equation (3), and pollution source inferred according to the water quality parameters expressed by factors.

| Parameters | Factor1 | Factor2 | Factor3 | Factor4 | Parameter weight（*W_i_*) |
| --- | --- | --- | --- | --- | --- |
| NO3-N | **0.908** | 0.147 | 0.128 | -0.101 | 0.0325 |
| IN | **0.834** | 0.43 | 0.154 | -0.13 | 0.0317 |
| Pe | **0.785** | 0.09 | 0.357 | 0.016 | 0.0353 |
| NO2-N | **0.755** | 0.24 | -0.157 | -0.04 | 0.0298 |
| CB | **0.7** | 0.127 | 0.086 | 0.175 | 0.0426 |
| TN | **0.652** | 0.047 | -0.031 | -0.218 | 0.0381 |
| FCB | **0.635** | 0.006 | -0.024 | 0.209 | 0.0430 |
| TP | **0.557** | 0.214 | -0.159 | 0.322 | 0.0311 |
| pH | **-0.555** | -0.055 | 0.091 | 0.434 | 0.0126 |
| AP | 0.196 | **0.929** | -0.038 | -0.07 | 0.0407 |
| N-NH3 | 0.251 | **0.879** | 0.157 | -0.034 | 0.0409 |
| Chl-a | 0.166 | **0.868** | 0.078 | -0.056 | 0.0421 |
| COD | -0.064 | **0.865** | -0.036 | -0.255 | 0.0367 |
| S | -0.294 | **-0.858** | 0.127 | 0.036 | 0.0067 |
| BOD5 | -0.058 | **0.799** | -0.162 | 0.127 | 0.0444 |
| NH3-N | 0.512 | **0.717** | 0.222 | -0.185 | 0.0375 |
| T | -0.424 | **0.64** | 0.094 | -0.326 | 0.0261 |
| Se | -0.144 | 0.054 | ***0.875*** | 0.164 | 0.0406 |
| MS | 0.422 | -0.051 | ***0.825*** | 0.153 | 0.0376 |
| Zn | -0.002 | -0.001 | ***0.723*** | -0.069 | 0.0241 |
| LAS | 0.305 | 0.224 | ***0.693*** | 0.017 | 0.0383 |
| Cd | -0.299 | 0.086 | ***0.68*** | 0.079 | 0.0336 |
| As | -0.163 | 0.119 | ***-0.632*** | 0.206 | 0.0300 |
| Ni | -0.322 | 0.204 | ***0.552*** | -0.054 | 0.0255 |
| TSS | -0.044 | 0.36 | ***-0.562*** | 0.272 | 0.0291 |
| T-Cr | -0.212 | 0.086 | -0.25 | ***0.675*** | 0.0330 |
| Hg | -0.07 | -0.072 | -0.031 | ***0.62*** | 0.0443 |
| SDD | -0.272 | 0.009 | 0.167 | ***0.613*** | 0.0121 |
| Pb | 0.012 | 0.08 | -0.161 | ***0.554*** | 0.0349 |
| Cu | -0.214 | 0.072 | -0.194 | ***-0.552*** | 0.0287 |
| DO | -0.263 | 0.14 | 0.037 | ***-0.584*** | 0.0165 |
| Factor weight (*W_i_*) | 0.2646 | 0.3177 | 0.2622 | 0.1555 |  |
| Pollution sources | Urban factors | Breeding /planting factors | Industrial factors | Other factors |  |

Note: The loading values in bold and italics in each column indicate that the corresponding water quality parameter is interpreted by the factor.
